# An In Silico Approach toward the Appropriate Absorption Rate Metric in Bioequivalence

**DOI:** 10.3390/ph16050725

**Published:** 2023-05-10

**Authors:** Vangelis D. Karalis

**Affiliations:** 1 Department of Pharmacy, School of Health Sciences, National and Kapodistrian University of Athens, 15784 Athens, Greece; vkaralis@pharm.uoa.gr; Tel.: +30-(210)-7274267; 2 Institute of Applied and Computational Mathematics, Foundation for Research and Technology Hellas (FORTH), 70013 Heraklion, Greece

**Keywords:** in silico methods, bioequivalence, pharmacokinetics, absorption rate, machine learning, Monte Carlo simulations, principal component analysis

## Abstract

In bioequivalence, the maximum plasma concentration (Cmax) is traditionally used as a metric for the absorption rate, despite the fact that there are several concerns. The idea of “average slope” (AS) was recently introduced as an alternative metric to reflect absorption rate. This study aims to further extend the previous findings and apply an in silico approach to investigate the kinetic sensitivity of AS and Cmax. This computational analysis was applied to the C-t data of hydrochlorothiazide, donepezil, and amlodipine, which exhibit different absorption kinetics. Principal component analysis (PCA) was applied to uncover the relationships between all bioequivalence metrics. Monte Carlo simulations of bioequivalence trials were performed to investigate sensitivity. The appropriate programming codes were written in Python for the PCA and in MATLAB^®^ for the simulations. The PCA verified the desired properties of AS and the unsuitability of Cmax to reflect absorption rate. The Monte Carlo simulations showed that AS is quite sensitive to detecting differences in absorption rate, while Cmax has almost negligible sensitivity. Cmax fails to reflect absorption rate, and its use in bioequivalence gives only a false impression. AS has the appropriate units, is easily calculated, exhibits high sensitivity, and has the desired properties of absorption rate.

## 1. Introduction

The goal of bioequivalence (BE) is to determine the in vivo equivalence of two pharmaceutical formulations containing the same active ingredient [1,2]. To achieve this goal, a pharmacokinetic (PK) comparison is performed between the two drug products, namely, the Test (T) vs. the originator’s product (Reference, R). After a certain statistical analysis, if the T formulation is shown to be pharmacokinetically equivalent to the R, it is also regarded as therapeutically equivalent. In other words, BE assessment is carried out in the context of pharmacokinetics, but the evidence of equivalence is projected into the therapeutic domain [3].

Officially, a T product is said to be bioequivalent to an R product if it contains the same active substance and there are no significant differences in absorption rate and amount when taken at the same dose as the R product [1,2,3]. The maximum plasma concentration (Cmax) is generally utilized to indicate absorption rate, while the area under the concentration-time (C-t) curve from time zero to the time of the last quantified concentration (AUC_0-t_ or, for simplicity just AUC) is used to describe the extent of absorption. Additional pharmacokinetic parameters are often utilized as secondary endpoints. The area under the C-t curve extrapolated to infinity (AUCinf) is also employed as the main endpoint for total exposure by the U.S. FDA [2]. Additional measurements include the time at which Cmax appears (i.e., Tmax) and the C-t curve terminal slope [1,2,3].

Regarding the extent of absorption, there are no doubts about using AUC as a measure expressing the degree of absorption. Despite the fact that Cmax has typically been employed as a metric of absorption rate in BE studies, it has been disputed as a metric that represents both the extent and rate of absorption [4,5,6]. Some studies have questioned the validity of Cmax as an absorption rate metric using modeling and simulation methodologies [7]. Other pharmacokinetic metrics, including the Cmax/AUC, Tmax, and partial AUCs, have been suggested in this context to address some of Cmax’s drawbacks [8,9,10,11,12,13,14,15,16,17,18]. Despite extensive research on other aspects of BE assessment (e.g., statistical framework, clinical designs), Cmax is widely used in BE studies worldwide.

Quite recently, two studies using machine learning (ML) approaches have appeared that debate the appropriateness of using Cmax as an absorption rate metric and introduce alternative measures [19,20]. In the first study, machine learning was employed for the first time in bioequivalence in order to investigate the relationships between the PK parameters and choose the best metric for absorption rate [19]. Using actual BE data as well as simulated datasets, it was shown that Cmax is not much related to absorption rate but mainly reflects the extent of absorption. The second study extended the findings of the previous work by introducing two new metrics for describing absorption rate: average slope (AS) and its weighted analog (ASw) [20]. Using a combination of population pharmacokinetics and machine learning algorithms, it was shown that AS and ASw are highly related to absorption rate. In addition, it was shown that the use of modern in silico approaches can assist in setting the appropriate parameters in BE testing. In addition, it should not be neglected that computational approaches have made significant contributions to bioequivalence and that numerous regulatory guidelines (e.g., highly variable drugs, adaptive designs, etc.) have been established using modeling and simulation methods [7].

This study moves one step further towards defining an appropriate measure for absorption rate in bioequivalence studies. The aim of this work is: (a) to verify the previous findings on the suitability of AS and ASw by applying ML to further explore their appropriateness in drugs with different absorption kinetics, and (b) to investigate the kinetic sensitivity of AS and ASw compared to the existing metrics (Cmax, AUC, and AUCinf); namely, to explore whether the desired performance seen from machine learning can also be observed in the field of pharmacokinetics. For the first goal of this study, principal component analysis (PCA) was selected as the most appropriate ML algorithm to identify the relationships between absorption rate and the pharmacokinetic metrics. For the second aim of the study, Monte Carlo simulations of joint pharmacokinetics–bioequivalence studies were performed to explore the kinetic sensitivity of all PK metrics. The aforementioned in silico approaches were applied to the kinetics of three drugs (hydrochlorothiazide, donepezil, and amlodipine), which exhibit different absorption kinetics.

## 2. Results

In this study, PCA was initially used to show the relationships between bioequivalence metrics, and in a second step, an in silico approach was applied to investigate the kinetic sensitivity of all these BE metrics. The assessment focused primarily on the comparative performance of Cmax and AS. The first metric, Cmax, refers to the currently proposed, by the regulatory authorities, absorption rate metric [1,2]. The second metric, AS, was recently introduced as a metric that successfully reflected the absorption rate characteristics [19,20].

**Figure 1:**
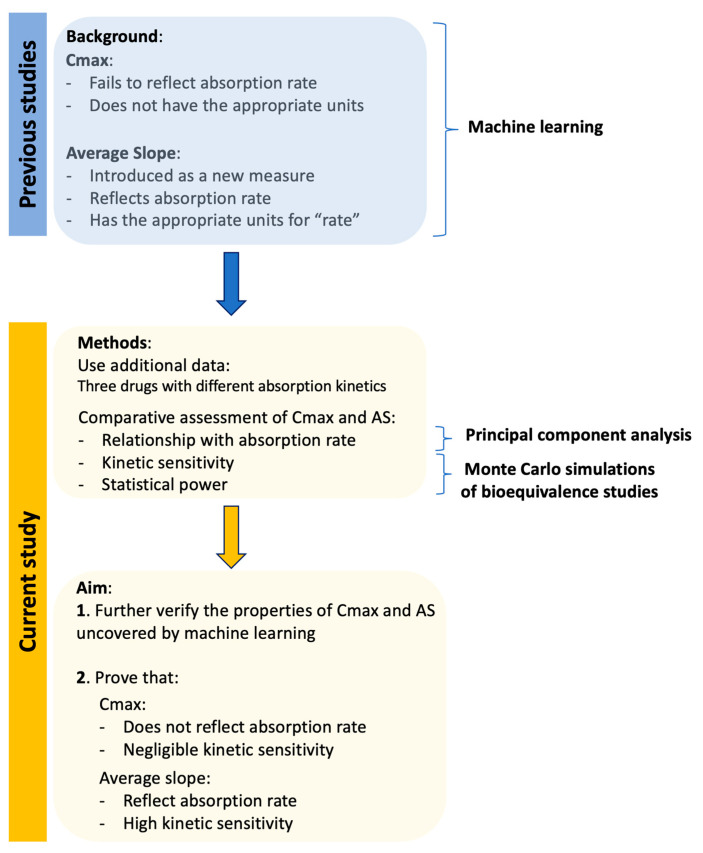
Outline of the analysis followed in this study to explore the properties of peak plasma concentration (Cmax) and average slope (AS).

The analysis started by estimating the PK variables of hydrochlorothiazide, donepezil, and amlodipine according to the strategy shown in Figure 1. The C-t data used in this study were generated using the PK models and model parameters reported in the literature [21,22,23,24,25,26]. For each drug, two-sequence, two-period, 2 × 2 crossover BE studies in 100 virtual subjects were generated. The BE metrics were calculated using non-compartmental techniques, as in actual practice. Then, the previously estimated PK data were subjected to principal component analysis.

### 2.1. Relationships between the Pharmacokinetic Parameters

**Figure 2:**
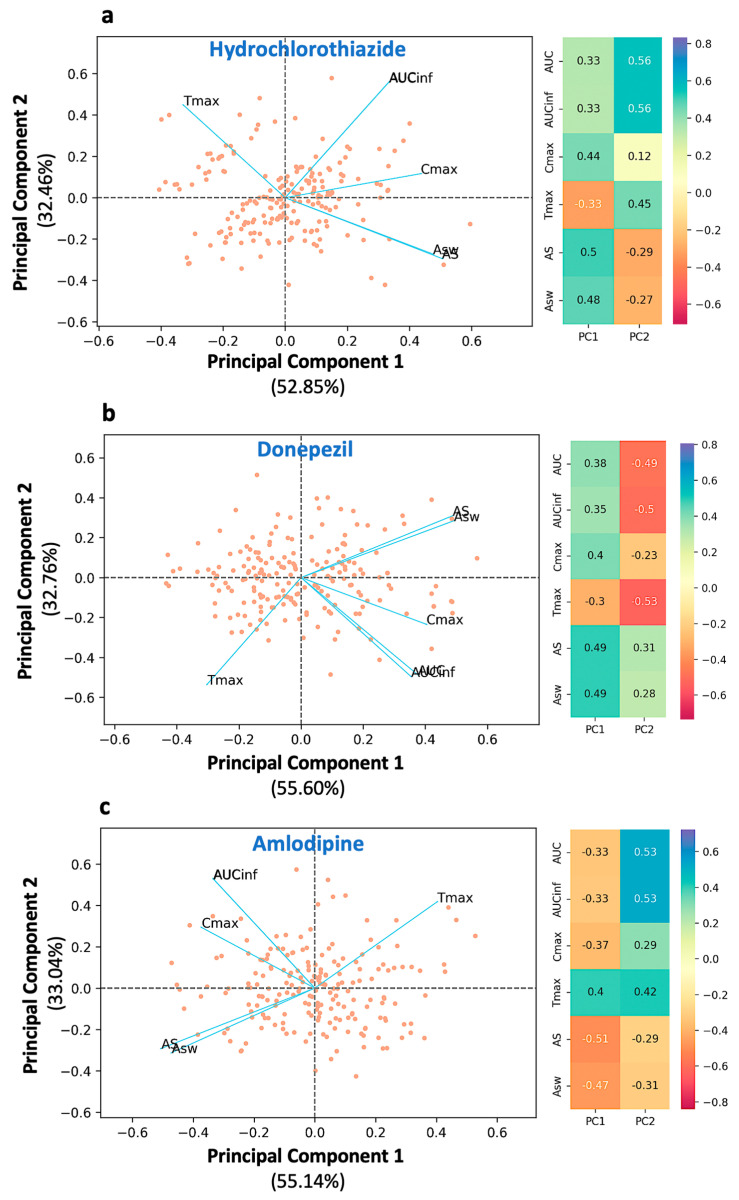
Principal component analysis of the bioequivalence metrics for the three drugs used in the study: (**a**) hydrochlorothiazide, (**b**) donepezil, and (**c**) amlodipine. Left panel: The biplot of the two principal components displaying individual scores (as dots) and the loadings of the bioequivalence metrics (as blue lines). Right panel: The loading values for each metric. Key: AS, average slope; ASw, weighted average slope; Cmax, peak plasma concentration; Tmax, the time at which Cmax is observed; AUC, area under the concentration-time curve up to the last quantifiable concentration; AUCinf, AUC extrapolated to infinity.

Initially, PCA was applied to further investigate the relationships between the PK parameters of drugs with different absorption kinetics. Figure 2 shows the PCA results for hydrochlorothiazide, donepezil, and amlodipine. The dots in the plane represent the observations (subjects in a BE study), while the lines reflect the vectors of the variables, such as Cmax, AS, ASw, AUC, AUCinf, and Tmax. Next to the PCA graphic (Figure 2) are the loadings (l1 and l2 for the first and second principal components) of all PK variables.

In all three cases, the same trend of findings can be observed. AS and ASw are adjacent to each other, both sharing almost the same loading values with respect to the first and second principal components (Figure 2). It should be highlighted that the AS (or ASw) vector is antidiametric to Tmax (the angle between them is close to 180°). For example, in the case of hydrochlorothiazide (Figure 2a), the angles of Tmax and AS with respect to PC1 are 126.2° and −30.1°, respectively. Thus, the angle ∠(AS·0·Tmax) is equal to their sum namely, 156.3°, close to 180°. This characteristic denotes the inverse kinetic behavior of AS and Tmax.

In other words, as AS increases, owing to quicker absorption, Tmax decreases and emerges at earlier time points. Thus, AS expresses the dynamic aspect of absorption. Similar findings were observed for the PCA plots of donepezil (Figure 2b) and amlodipine (Figure 2c). AUC and AUCinf are superimposed and, plausibly, both share the same l1 and l2 values. The two AUC terms are located almost perpendicular to the Tmax axis. The angle ∠(AUC·0·Tmax) is close to 90°, which indicates the weak relationship between AUC and absorption rate. For example, in the case of hydrochlorothiazide, the angle formed between the AUC and Tmax vectors is 66.7°, i.e., close to orthogonal. Similar findings can also be observed in the case of amlodipine.

In all three plots (Figure 2a–c), the vector of Cmax is found between that of AS and AUC. This implies that Cmax is not completely independent from absorption rate, as AUC is, but it has a weak association with it. However, the angle formed between Cmax and AS, namely ∠ (Cmax·0·AS) is much smaller than that of ∠(Cmax·0·AUC) implying that Cmax mostly expresses the extent of absorption rather than rate. For example, in the case of hydrochlorothiazide (Figure 2a), the angle ∠(Cmax·0·AUC) is 44.2°, while the angle between Cmax and the opposite direction of Tmax ∠(Cmax·0·−Tmax) is 69.0°. Thus, Cmax exhibits a much higher relationship with the extent of absorption (i.e., AUC) than the rate of absorption (i.e., Tmax or equivalently −Tmax). It is worth mentioning that the same results are observed in all three drugs regardless of their different absorption kinetic properties. The latter indicates the consistency of the findings, namely, the association between the PK metrics.

**Figure A1:**
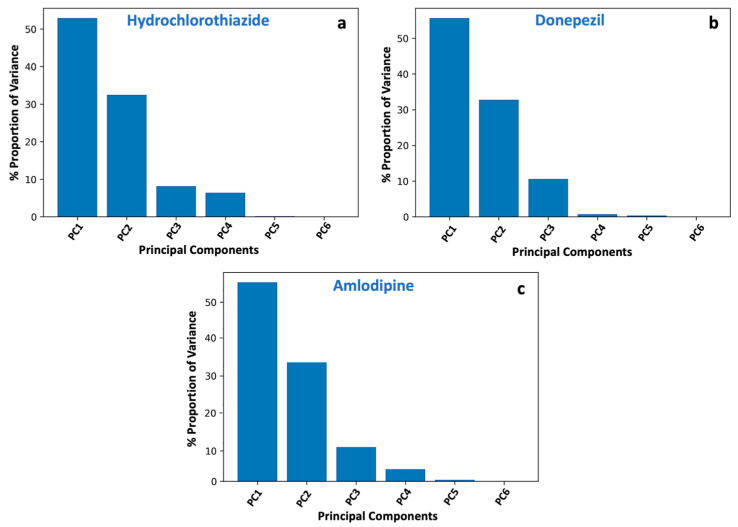
Scree plots to define the number of principal components (PC) for the PCA models of hydrochlorothiazide (**a**), donepezil (**b**), and amlodipine (**c**).

The desired descriptive ability of the developed PCA models is reflected in the high values of the explained variance. For hydrochlorothiazide, the first and second principal components were found to account for 85.31% of the total variability (52.85% and 32.46% of the overall variability, respectively). For donepezil, the total explained variability was 88.36%, while for amlodipine, it was found to be 88.18%. Scree plots were developed in order to determine the ideal number of main components for the PCA models of hydrochlorothiazide, donepezil, and amlodipine (Figure A1). The *y*-axis displays the eigenvalues (percent of variance explained), while the *x*-axis displays the number of components. The scree plot criterion looks for the “elbow” curve and selects all components just before the line flattens out.

### 2.2. Pharmacokinetic–Bioequivalence Simulations

#### 2.2.1. Sensitivity

**Figure 3:**
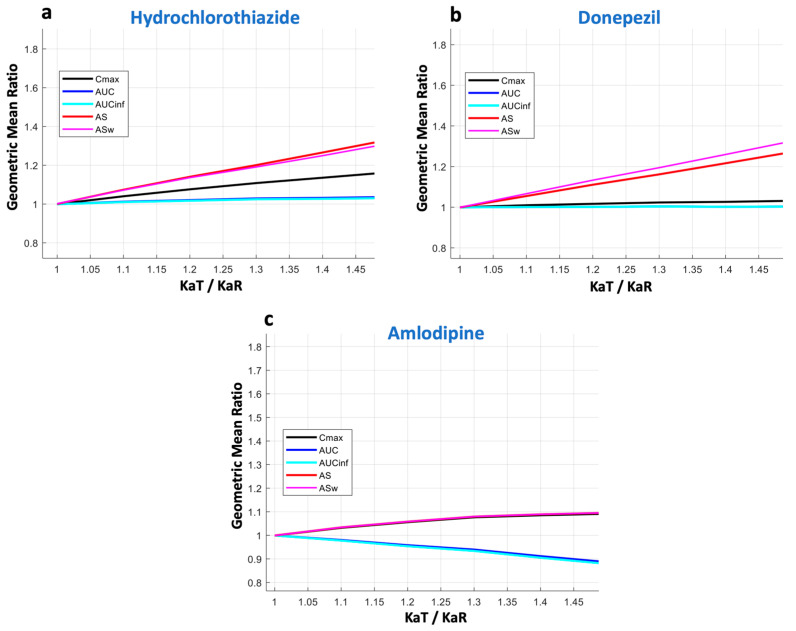
Kinetic sensitivity of the bioequivalence metrics. The vertical axis refers to the geometric mean ratio of each metric, while the horizontal axis is the ratio of the absorption rate constants (KaT/KaR) of the test formulation over the reference formulation. Key: AS, average slope; ASw, weighted average slope; Cmax, peak plasma concentration; Tmax, the time at which Cmax is observed; AUC, area under the concentration-time curve up to the last quantifiable concentration; AUCinf, AUC extrapolated to infinity.

In this study, the kinetic sensitivity of the PK metrics was assessed through the application of PK-BE simulations. Figure 3 presents the relationship between the GMR of each bioequivalence metric (i.e., Cmax, AUC, AUCinf, AS, and ASw) as a function of the T/R ratio of absorption rate (i.e., KaT/KaR). In all these simulations, the KaR value was kept constant, equal to the literature value, while the KaT value was allowed to change. Simulations were repeated for each KaT/KaR ratio, and the GMR of each BE metric was recorded. Since at each KaT or KaR value, 10,000 Monte Carlo trials were simulated, the presented GMR estimate refers to the average GMR from these simulation trials.

In the case of hydrochlorothiazide (Figure 3a), it becomes obvious that there is a linear increase in the GMR of AS. A similar performance can be seen for ASw, with its increase being slightly steeper. The latter is expected due to the nature of ASw, which is the weighted version of AS, and the fact that more emphasis is placed on early time points that are affected more by changes in the absorption rate. For Cmax, only a subtle increase can be observed; namely, the slope of the curve is much smaller compared to that of AS or ASw. This finding shows the lower kinetic sensitivity of Cmax compared to AS and ASw. As expected, AUC and AUCinf remain almost unaltered with the changes in absorption rate.

A similar pattern was observed for donepezil (Figure 3b). Again, AS and ASw were the most sensitive metrics to reflect changes in absorption rate. Conversely, Cmax appeared to be almost non-responsive to changes in absorption rate and showed a behavior similar to AUC (and AUCinf). Donepezil has a lower absorption rate compared to hydrochlorothiazide, and it appears that Cmax completely failed to reflect the changes in the speed of absorption.

The last example was that of amlodipine (Figure 3c), which is absorbed much more slowly (Tmax lies between 6 and 12 h) than the other two drugs (hydrochlorothiazide and donepezil). Again, in this case, AS and ASw succeed in reflecting the absorption rate changes, while all other metrics fail.

**Figure A2:**
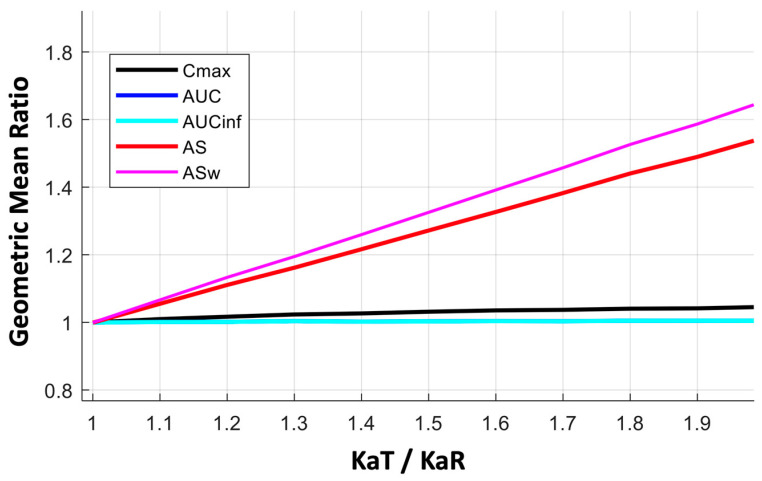
Kinetic sensitivity of the bioequivalence metrics for a wide range (from 1.0 to 2.0) of the absorption rate constants ratio (KaT/KaR) in the case of donepezil. Key: AS, average slope; ASw, weighted average slope; Cmax, peak plasma concentration; Tmax, the time at which Cmax is observed; AUC, area under the concentration-time curve up to the last quantifiable concentration; AUCinf, AUC extrapolated to infinity.

In the three cases shown in Figure 3, the KaT/KaR ratio ranges from complete similarity (i.e., when it is equal to 1) up to a 50% difference (i.e., KaT/KaR = 1.5). The performance of all BE metrics over a wider range of absorption rates (up to 100% change, i.e., KaT/KaR = 2) is depicted in Figure A2 in the case of donepezil. The results of Figure A2 are in line with those shown in Figure 3, where the high sensitivity of AS and ASw can be contrasted to the almost negligible sensitivity of Cmax, even for differences in absorption rates as high as 100%. That means that practically, Cmax cannot reflect the absorption rate.

#### 2.2.2. Statistical Power

**Figure 4:**
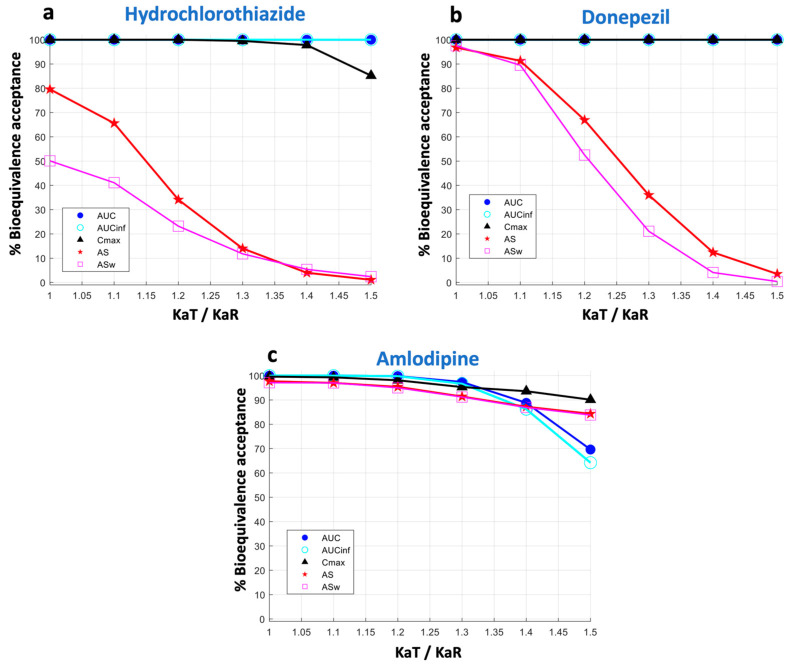
Statistical power of the bioequivalence metrics. The vertical axis refers to the percent of bioequivalence acceptance and the horizontal axis is the ratio of the absorption rate constants (KaT/KaR) of the test formulation over the reference formulation. Key: AS, average slope; ASw, weighted average slope; Cmax, peak plasma concentration; Tmax, the time at which Cmax is observed; AUC, area under the concentration-time curve up to the last quantifiable concentration; AUCinf, AUC extrapolated to infinity.

Since both AS and ASw metrics showed high kinetic sensitivity to absorption rate, it is expected that this would be reflected in their statistical power to declare bioequivalence. Therefore, the simulation methodology also captured the BE acceptance rate of each metric as a function of the KaT/KaR ratio. Again, the percent BE acceptance at each KaT or KaR point comes from 10,000 simulated trials performed in each case. Figure 4 summarizes these results for statistical power in the cases of hydrochlorothiazide (Figure 4a), donepezil (Figure 4b), and amlodipine (Figure 4c). As it was anticipated, the highest sensitivity of ASw is reflected in the lower probability of acceptance compared to all other metrics. AS comes next, which is more permissive compared to ASw but shows a lower probability of acceptance compared to Cmax, AUC, and AUCinf. This attribute becomes more evident as the absorption rate of a drug increases, i.e., when a drug is absorbed faster. Indeed, the reduction in the statistical power of AS and ASw appears more pronounced in the case of the rapidly absorbing hydrochlorothiazide (Figure 4a). For donepezil (Figure 4b), the reduced percentages of acceptances for AS and ASw are also observed, but this attribute becomes smaller. However, for a slowly absorbing drug like amlodipine (Figure 4c), the permissiveness of AS and ASw becomes quite similar to that of Cmax and AUC.

This reduction in the statistical power observed in the AS and ASw is expected and reflects the combined action of two factors: (a) the higher discrepancy in the GMR estimates (see Figure 3), and (b) the higher variability that accompanies the estimation of the AS and ASw. The first reason is a desired feature and reflects the high kinetic sensitivity of AS or ASw, namely, their inherent ability to capture absorption rate. The second reason originates from the estimation methods of AS and ASw (Equations (1) and (2) in “Section 4”). Both equations actually represent the highly dynamic conditions of the kinetics that occur during the absorption process. In other words, absorption rate is not a stable process, but it changes continuously and therefore high variability is observed.

**Figure 5:**
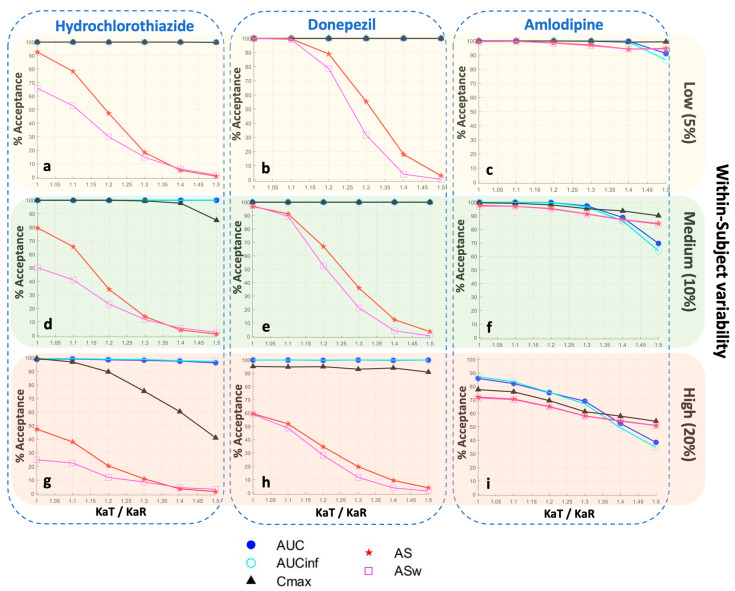
Statistical power of the bioequivalence metrics for three different levels of within-subject variability in each of the model parameters: low (5%), medium (10%), and high (20%). In each plot, the vertical axis refers to the percent of bioequivalence acceptance, while the horizontal axis is the ratio of the absorption rate constants (KaT/KaR) of the test formulation over the reference formulation. Key: AS, average slope; ASw, weighted average slope; Cmax, peak plasma concentration; Tmax, the time at which Cmax is observed; AUC, area under the concentration-time curve up to the last quantifiable concentration; AUCinf, AUC extrapolated to infinity.

In order to explore the impact of variability on the permissiveness of the BE metrics, Figure 5 was constructed. Three different levels of within-subject variability in each of the model parameters were used: low (5%), medium (10%), and high (20%). A visual inspection of Figure 5 reveals that as within-subject variability increases, the statistical power of AS and ASw decreases. This effect is more evident for a rapidly absorbing drug, like hydrochlorothiazide (Figure 5a,d,g), while it becomes less pronounced as the drug is absorbed more slowly; namely, it is less noticeable for donepezil (Figure 5b,e,h), while all BE measures appear to be affected to the same extent in the case of amlodipine (Figure 5c,f,i), which is a slowly absorbed drug.

**Figure 6:**
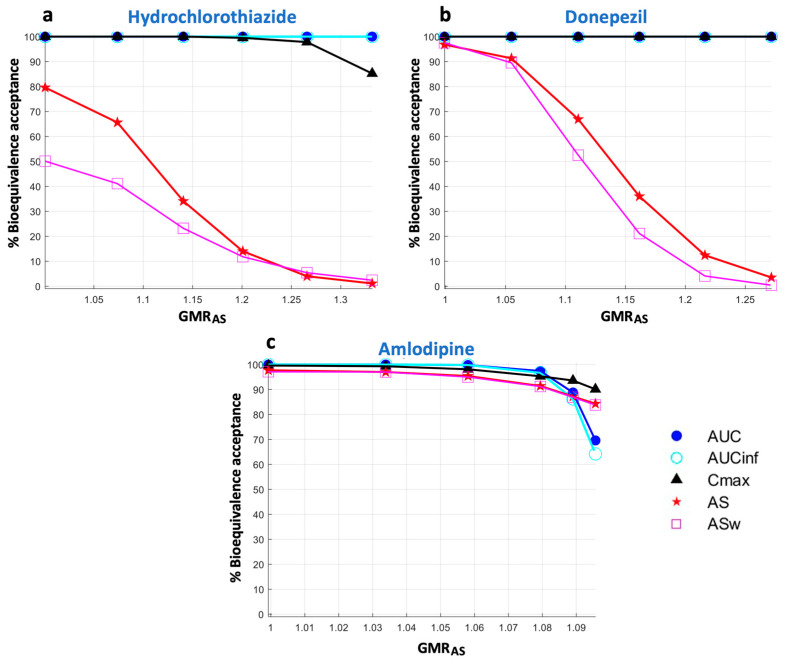
Statistical power of the bioequivalence metrics as a function of the geometric mean ratio of average slope (GMR_AS_). Key: AS, average slope; ASw, weighted average slope; Cmax, peak plasma concentration; Tmax, the time at which Cmax is observed; AUC, area under the concentration-time curve up to the last quantifiable concentration; AUCinf, AUC extrapolated to infinity.

It is worth mentioning that the high sensitivity of AS and ASw, which is accompanied by a reduction in their statistical power, can also be depicted with changes in the GMR of AS (or ASw respectively) (Figure 6). These findings are depicted in Figure 6, which shows the percent acceptances of all BE metrics as a function of GMR_AS_. In line with the findings derived from Figure 4, it is also shown that the reduction in statistical power is more evident as the absorption kinetics of a drug becomes faster. Namely, there appears to be a minimal reduction for amlodipine (Figure 6c), while this attribute increases for donepezil (Figure 6b) and is much more evident in the case of hydrochlorothiazide (Figure 6a).

## 3. Discussion

The aim of this study was to find an appropriate metric for the rate of absorption. For this reason, a deeper look into the features of the two recently introduced absorption rate metrics (AS and ASw) was made [19,20]. In this context, initially, the recent findings of the machine learning approaches were further extended and verified, regarding the suitability of AS and ASw and the failure of Cmax to reflect absorption rate. The superior kinetic sensitivity of the two new metrics (AS and ASw) was demonstrated when they were compared against that of Cmax and the two other metrics (AUC and AUCinf) for expressing the extent of absorption.

Initially, PCA was used to explore the relationships between the PK parameters of the three drugs. The high values of the percent explained variance for hydrochlorothiazide, donepezil, and amlodipine (85.31%, 88.36%, and 88.18%, respectively) demonstrate the expected high descriptive ability of the developed PCA models. The PCA findings indicated almost similar performance for all three drugs (Figure 2). AS and ASw are adjacent to one another and inversely proportional to Tmax (the angle between them is close to 180°). This property denotes the inverse kinetic behavior of AS and Tmax. In other words, as AS increases, owing to quicker absorption, Tmax decreases and emerges at earlier time points. As a result, AS is successful in depicting the dynamic aspect of absorption. AUC and AUCinf are stacked, with both terms approximately perpendicular to the direction of AS-Tmax. This discovery emphasizes the tenuous link between AUC and absorption rate. The Cmax vector is found between the AS and AUC vectors. This shows that, unlike AUC, Cmax is not fully independent of absorption rate but has a tenuous relationship with it.

A visual inspection of Figure 2 reveals that the absolute positions of the PK vectors are not the same. However, the relative positions between them are rather similar. This attribute is expected due to the fact that drugs with different absorption kinetic attributes were used and a rotation setting was utilized in the PCA analysis, which resulted in differently adjusting the vectors in the two-dimensional space. The results of all analyses agree that Cmax is only slightly related to absorption rate, while AS and ASw are almost perfect reflections of it. In a previous study, the PCA analysis was performed using additional PK parameters (e.g., Cmax/AUC, terminal slope, average concentration, Cmax/Tmax) to those utilized in this analysis [20]. The reason why this study focused only on the selected parameters (AS, ASw, Cmax, Tmax, AUC, and AUCinf) is that it was intended to avoid adding variability through the other metrics and to isolate the performance of the measures of absorption rate and extent. In other words, it allowed for a clearer understanding of the properties and relationships of the parameters under investigation. Nevertheless, the findings observed in the current analysis are in full agreement with those reported in the previous study [20].

For the purposes of this study, N was set equal to 12, 24, and 48 in the case of Monte Carlo simulation studies, while a sample size of N = 100 was used for the PCA. The latter was set large enough to allow reliable application of the PCA. Even though a sample size of 100 is rarely used in BE studies, it was applied in this study for the purposes of machine learning. In addition, due to the fact that 100 virtual subjects were simulated in a 2 × 2 crossover study, the total number of records in PCA was doubled, namely, 200, because there is no reason to discriminate between the T and R formulations.

The analysis continued with the investigation of the kinetic sensitivity of all metrics. If a change in one of the examined kinetic parameters results in an equivalent change in the measured metric, the latter is said to have full kinetic sensitivity [27]. With regard to the rate of absorption, full kinetic sensitivity would prevail if, for example, a 25% change in the latter would result in a 25% change in the absorption rate metric. If, however, a change in the parameter produces a considerably smaller change in the metric, the metric lacks full kinetic sensitivity to the parameter [27]. In this study, this investigation task was achieved by applying a Monte Carlo pharmacokinetic–bioequivalence simulation approach. This in silico method showed that the most sensitive measures to indicate changes in absorption rate were AS and ASw (Figure 3). Conversely, Cmax appeared to be practically non-responsive to variations in absorption rate and exhibited behavior comparable to AUC. The same pattern was observed for all three drugs, and it was more evident as the absorption process of a drug is faster (Figure 3a). In the case of hydrochlorothiazide, a 50% difference in absorption rate leads to 33.2% and 32.1% differences in AS and ASw, respectively. However, for Cmax, the difference was only 16.3%, implying a rather poor sensitivity. For donepezil (Figure 3b), which is absorbed moderately fast, AS and ASw kept their high sensitivities (27.2% and 32.5%, respectively), while Cmax showed only a 3.1% increase as a result of a 50% change in absorption. In addition, it should be underlined that the kinetic sensitivity results are in line with the PCA findings (Figure 2). Actually, the results from these two different approaches complement each other and allow for a broader understanding of the characteristics of an absorption rate metric.

The findings regarding kinetic sensitivity found in this study are in agreement with the study of Vincze et al., who studied the kinetic sensitivities of Cmax and partial AUCs under single and multiple administration [27]. It was shown that Cmax was less sensitive even when compared to partial areas. In addition, it should not be disregarded that even from the beginning of its use, Cmax was considered a supplementary measurement of the extent of absorption following AUC [28]. In bioequivalence research, Cmax can only serve as an indication of absorption rate. For this reason, many studies have tried in the past to find appropriate measures for characterizing absorption rate [8,10,11,13,14,17].

The use of Cmax as a measure of absorption rate has created various issues [4,5,6,10,12,13]. Other metrics based on in silico approaches have been proposed in this context to define the best absorption rate metric. Tmax and the Cmax/AUC ratio have been presented as suitable replacements for Cmax for analyzing the absorption rate characteristics of immediate-release formulations [6]. Tmax and the Cmax/AUC ratio share similar characteristics, according to studies comparing the absorption rates of two drug formulations [6]. This finding offered persuasive evidence for adopting the observed Cmax/AUC ratio as a measure of absorption rate rather than Tmax since Cmax/AUC was easier to handle statistically and could be assessed with more precision than Tmax [6,10]. However, the consideration of a parameter as a measure of absorption rate is grounded in a fundamental theoretical rationale. The latter pertains to the system of quantification [20]. The utilization of a parameter to denote the “rate” of a process necessitates the representation of the alteration of a measure, such as distance or concentration, with respect to time. When considering absorption, a suitable metric for measuring absorption rate should be expressed in units of concentration per time. This implies that the aforementioned metric ought to be computed as the quotient of changes in drug concentration values and the corresponding temporal changes. It is apparent that except for AS (and ASw), all other metrics suggested in the literature do not possess suitable units. The various representations of the areas under the curves, namely AUC, AUCp, and AUCinf, pertain to geometric areas and are quantified in terms of concentration multiplied by time units. Similarly, Cmax and Tmax do not exhibit the appropriate units. The utilization of the Cmax/AUC ratio has also been suggested as a substitute for Cmax with the objective of eliminating the impact of absorption extent. This is achieved by dividing Cmax with AUCt. Nevertheless, the Cmax/AUC ratio does not have the appropriate nature since it has units of time^−1^.

Another important issue is the simplicity and applicability of a pharmacokinetic measure. AS and ASw can be calculated using non-compartmental methods, a necessary prerequisite for the simplicity and reproducibility of the estimations. In this vein, model-based approaches are not suitable for BE assessment because the settings used, the assumptions made, the appropriateness of model fitting, and algorithm convergence all affect them. On the contrary, AS (or ASw) can be easily estimated without any model assumptions or any other prerequisites [20]. The calculation of the average slope is quite simple and can be made without the use of any sophisticated software. It is so easy that it can be implemented in an Excel^®^ spreadsheet directly from the raw C-t data. For this reason, an Excel^®^ file performing the estimation of the basic pharmacokinetic parameters, including AS, can be found in the Supplementary Materials.

It is worth mentioning that, in this study, the in silico approach was implemented in MATLAB^®^. Nevertheless, other programming languages, such as Python or R, could have been used. In addition, the simulations could have been performed in software oriented toward pharmacokinetics, such as Simulx^TM^ (Monolix^TM^) or NONMEM^®^. The reason for using MATLAB^®^ relies on its previous use in the development of the IVIVS technique [21]. It should be underlined that for the use of AS (or ASw) in BE assessment, it is not necessary to use MATLAB^®^, but the estimation of AS can be easily made in any software using a simple non-compartmental approach. As mentioned above, the calculations are so easy that they can be made in Excel^®^.

Because both the AS and ASw measures demonstrated strong kinetic sensitivity to absorption rate, it was therefore hypothesized that their statistical power to declare bioequivalence would be affected. As a result, the simulation methodology captured also the percentage BE acceptability of each statistic as a function of the KaT/KaR ratio. The high sensitivity of ASw was reflected in the lowest probability of acceptance when compared to the other metrics (Figure 4). AS comes next, which was more permissive than ASw, but had a lesser chance of acceptance than Cmax, AUC, and AUCinf. This characteristic becomes more apparent as a drug absorption rate increases, i.e., when a drug is absorbed faster. In a rapidly absorbed drug, like hydrochlorothiazide, when the absorption rate differs by 20%, the percent BE acceptance according to AS would be 34.1%, for ASw 23.2%, but the Cmax metric would be completely insensitive, and the permissiveness would stay at 100% (Figure 4a). Even for much greater discrepancies, namely a 50% difference in the absorption rate, the corresponding percentages of acceptance would be 1.1%, 2.4%, and 85.2% for AS, ASw, and Cmax, respectively. The latter simply implies that Cmax completely fails to conceive changes in absorption rate. Similar findings can be observed for donepezil, which is a moderately fast at being absorbed drug (Tmax = 3–4 h). In the case of donepezil, and assuming a 30% difference, the corresponding BE acceptances are 36%, 21.1%, and 100% for AS, ASw, and Cmax, respectively (Figure 4b). The same trend was observed for the slowly absorbed drug, amlodipine (Figure 4c).

**Figure A3:**
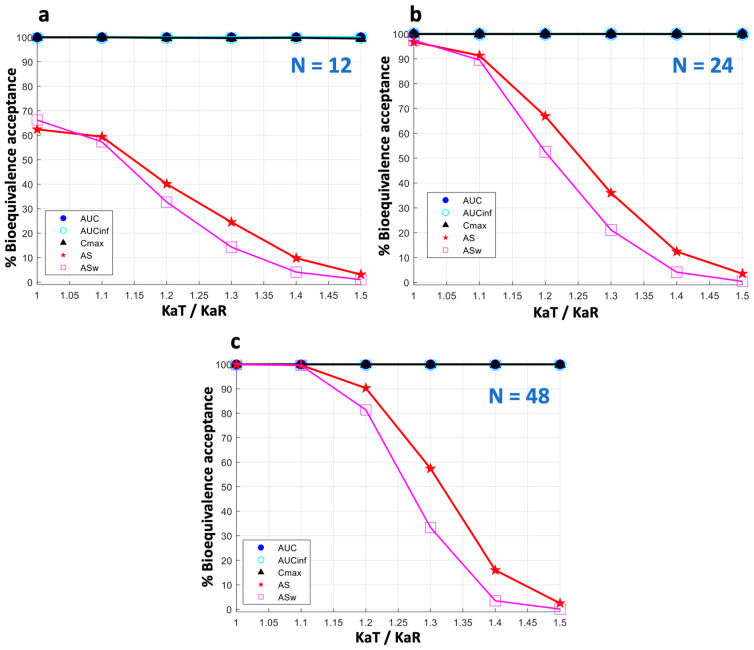
The impact of sample size (N) on bioequivalence acceptance of donepezil. Three sample size levels are shown: (**a**) N = 12, (**b**) N = 24, (**c**) N = 48.

**Figure A4:**
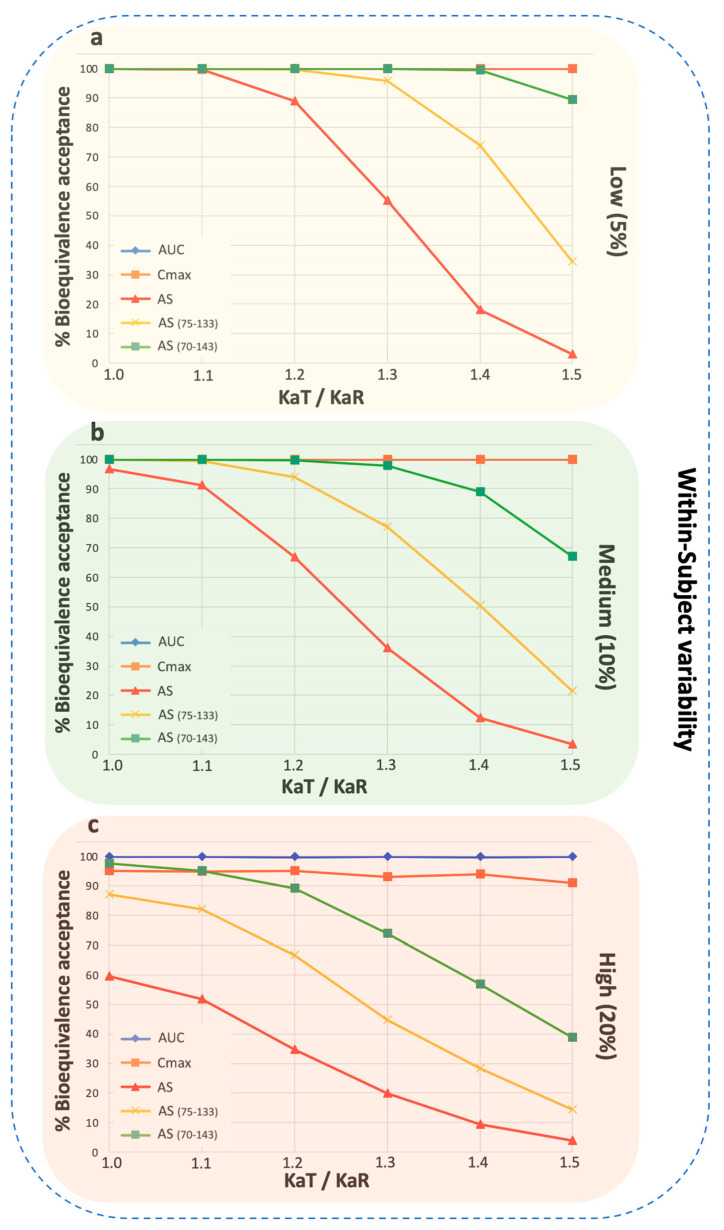
Percent bioequivalence acceptance for donepezil when the wider acceptance limits are used in the case of average slope. (**a**) hydrochlorothiazide, (**b**) donepezil, and (**c**) amlodipine. The wider limits refer to 75–133% and 70–143% [29]. Three different levels of within-subject variability are shown: low (5%), medium (10%), and high (20%). Key: AS, average slope; ASw, weighted average slope; Cmax, peak plasma concentration; Tmax, the time at which Cmax is observed; AUC, area under the concentration-time curve up to the last quantifiable concentration; AUCinf, AUC extrapolated to infinity.

Actually, the reduced statistical power of the AS and ASw estimates represents the combined action of the larger disparity in the GMR estimates (see Figure 3) and the higher variability associated with the AS and ASw estimates. The first, the bigger difference in the GMR estimates of AS and ASw, is a desirable characteristic that shows AS and ASw exhibit strong kinetic sensitivity. The high within-subject variability of these two metrics reflects the extremely dynamic conditions that occur during the absorption process. In other words, the absorption rate is a dynamic process that changes constantly. Possible ways to increase the statistical power of AS and ASw can include the use of larger sample sizes (Figure A3) and/or the widening of acceptance limits (Figure A4). Figure A4 demonstrates the increase in the percent acceptances when the acceptance limits are set to be wider, for example, equal to those proposed in the previous regulatory guidelines, such as 75–133% and 70–143% [29]. In the same context, increasing the sample size could also lead to increases in permissiveness (Figure A3).

It should be clarified that increasing the sample size is not proposed in this study as a solution for increasing the statistical power; only its impact on the percent BE acceptance. As with any clinical trial, ethical concerns should always take precedence. Other approaches, like the use of scaled limits, as in the case of highly variable drugs, or the widening to constant pre-fixed values (e.g., 70–143%), can provide solutions. Nevertheless, it should be underlined that the decreased statistical power observed from the use of AS (or ASw) is anticipated by the nature of these metrics since they are quite sensitive to differences in the absorption rate. The important issue is that AS (or ASw) is a metric that distinctly expresses absorption rate, and by performing additional modeling and simulation work, we can make the necessary adjustments to increase its statistical power to an acceptable level. It is in the hands of regulatory authorities to choose and set up the appropriate bioequivalence acceptance limits for AS or ASw.

It was mentioned above that the decrease in the probability of acceptance of AS and ASw refers to the actual changes in the absorption rate and not the GMR estimate of the metric. Figure 6 shows the performance of AS and ASw (and all other metrics) when the statistical power is plotted as a function of the GMR of AS (i.e., GMR_AS_). In this case, as expected, a 25% difference in the GMR_AS_ estimate leads to a 5% acceptance for AS (Figure 6a,b). Again, the same pattern is observed, with ASw being the most sensitive and thus exerting the lowest BE acceptance, followed by AS, while Cmax did not respond to the changes of GMR_AS_. Thus, there is no inflation of type I errors by using either AS or ASw.

Generally, in this study, the appropriateness of Cmax as an absorption metric in BE studies is investigated and strongly questioned. Both the PCA and the simulation approaches coincide and show clearly that Cmax cannot reflect the rate of absorption and is completely insensitive. Conversely, the two newly proposed metrics, AS and ASw, succeed in both situations. Overall, AS (or ASw) can be simply calculated from the C-t data and has concentration/time units, unlike all other absorption rate metrics, which have meaningless units. The ML approaches showed that AS (or ASw) accurately reflects “absorption rate”, while Cmax and other metrics fail. The kinetic sensitivity analysis proved their inherent characteristics easily reflect changes in the absorption rate in a linear trend (Figure 3), either for slowly, moderately, or quickly absorbed drugs. Conversely, Cmax, which is the traditionally used metric, fails to express the kinetic properties of Cmax (see Figure 2 and the previous studies [19,20]), does not have the appropriate units of “rate”, and as a consequence has negligible sensitivity to detect differences in absorption rate [19,20]. Other metrics that have been proposed in the literature, like Cmax/AUC or partial AUC, were proven in the two previous studies using machine learning approaches to not exhibit the desired properties for expressing the rate of absorption [19,20]. Conclusively, the advent of machine learning allows the investigation of an old problem from a new perspective and the offering of new solutions.

In addition, the in silico methodology with Monte Carlo simulations used in this study allowed more realistic predictions since it can describe the kinetics of any drug using the appropriate systems of ordinary differential equations (e.g., one-, two-, three-compartment models, with or without lag times, etc.) and it considers between- and within-subject variabilities, actual sampling schemes, the generation of any number of virtual subjects, adapting them appropriately in a 2 × 2 crossover clinical design, and perform any number of trials (e.g., 10,000 trials in each case in this study). Also, the choice of drugs used in this study was based on their different absorption kinetic properties. Hydrochlorothiazide is a drug that is absorbed rapidly after oral administration (Tmax: 1.5–2 h), while donepezil is absorbed more slowly (Tmax: 3 h), and amlodipine even more slowly (Tmax: 6–12 h). Their disposition is also different since hydrochlorothiazide is described by a two-compartment model, whereas donepezil and amlodipine are described by one-compartment models. This diversity of kinetics allowed for exploring the properties of AS and other metrics under different conditions.

The appropriateness of an absorption metric is quite important in bioequivalence studies since it is one of the endpoints that are being used in the statistical assessment of bioequivalence. For AUC, there is no doubt that it expresses the extent of absorption; however, for Cmax, there has always been the belief that it does not express absorption rate, but it was used since no other better metric existed. In strict terms, Cmax was actually considered a measure to express “peak exposure”, rather than absorption rate [1,4,16].

A limitation of this study is that the PCA and the Monte Carlo simulation approaches have been performed for three drugs. Even though these three drugs were selected to have different absorption kinetics, thus allowing them to be explored under different conditions, more studies on drugs with different absorption profiles would be beneficial. The proposed metrics, AS and ASw, are intended to be used for assessing the absorption rate under single-dose administration. Their application after multiple dosing, as in the case of modified-release products, has not been studied. However, the estimation method of AS (or ASw) does not prohibit its use in the steady state. Therefore, future research can focus on drugs from different routes of administration (e.g., inhalation), drugs with complex absorption kinetics (e.g., parallel, transit compartments), bioequivalence studies in the fed state, and after multiple dosing.

## 4. Materials and Methods

### 4.1. Route of Analysis

In order to facilitate the understanding of the methodology used in this study, an outline of the route of analysis is illustrated in Figure 1. Previous studies using machine learning approaches have shown that Cmax not only lacks the appropriate units to express “rate” but also fails to reflect absorption rate. The average slope and its weighted analog were introduced to be in accordance with the theoretical expectations to express “rate” and this attribute was proved using ML.

This study expands the previous work by exploring the relationships of all PK metrics using ML for three drugs with different absorption properties. In addition, and most importantly, this study explores the kinetic sensitivity of AS and ASw, compared to Cmax and other PK metrics, using Monte Carlo simulations under several pharmacokinetic scenarios.

### 4.2. Drugs and Pharmacokinetic Properties

Three drugs with different absorption kinetics were used in this analysis. The main pharmacokinetic characteristics of hydrochlorothiazide, donepezil, and amlodipine are summarized in Table 1.

Hydrochlorothiazide is a thiazide diuretic that is extensively used in adults and children to treat hypertension and edema caused by fluid overload [30,31]. With a pKa value of 7.9, hydrochlorothiazide belongs to Biopharmaceutics Classification System (BCS) class II and is extremely permeable, with low water solubility and weak base characteristics [32]. It belongs to BCS subclass IIb, and its solubility in stomach contents differs greatly from that in the small intestine [33,34]. It is predicted to be delayed after oral delivery since it must reach the small intestine to be digested and absorbed. As a result, numerous other processes occur prior to absorption, including reduced dissolution and precipitation in the stomach, gastric emptying, transit in the small intestine, and intestinal content dissolution [33]. The absorption of hydrochlorothiazide ranges from 60% to 80%, with the majority occurring in the duodenum and upper jejunum following stomach dissolution [30,31]. The pharmacokinetics of hydrochlorothiazide are linear throughout a dosage range of 5–100 mg. Maximal plasma concentration occurs within 1 to 1.5 h with a small lag time [30,31].

Donepezil is used to treat the symptoms of mild to moderately severe Alzheimer’s disease [35]. Donepezil inhibits the acetylcholinesterase enzyme, which typically degrades acetylcholine, in a selective and reversible manner. The treatment is started at 5 mg/day (once-a-day dosing). Donepezil is well absorbed and has a 100% relative oral bioavailability, reaching peak plasma concentrations in 3–4 h [35]. The pharmacokinetics of 1–10 mg given once a day are linear. The BCS categorization of donepezil is not established in the literature. According to recent research, donepezil is a BCS I medication [36].

The third medicine, amlodipine, was chosen since it is slowly absorbed [37]. Amlodipine is a calcium channel antagonist with a long half-life that selectively blocks calcium ion influx across cell membranes [37]. It is extensively metabolized in the liver (with no appreciable presystemic or first-pass metabolism) and removed slowly, having a terminal elimination half-life of 40–50 h. Amlodipine is well absorbed after oral administration, with peak blood levels occurring 6–12 h after treatment. The absolute bioavailability ranges between 64 and 80% [37].

### 4.3. Pharmacokinetic-Bioequivalence Metrics

The pharmacokinetic metrics investigated in this study are the following:

- AUC and AUCinf as metrics to express the extent of absorption
- Cmax, which is the traditionally used metric for assessing absorption rate
- AS and ASw, referring to the average slope and its weighted analogue

**Figure 7:**
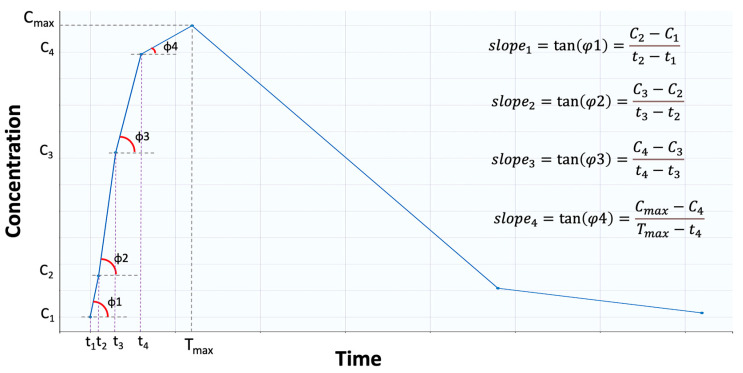
Graphical representation of the “average slope”. Average slope is the average of the tangents (i.e., slopes) formed between two consecutive points. In this example, four points are shown before the maximum concentration (Cmax) is reached.

The estimation formula for average slope is given by Equation (1), and it refers to the mean of all sequential slopes (Figure 7), between the concentrations and times, up to Tmax [20]:

(1) $$\mathrm{AS}=\frac{\sum_{i=1}^{n-1}slope_{i}}{n-1}=\frac{\sum_{i=1}^{n-1}\frac{C_{i+1}-C_{i}}{t_{i+1}-t_{i}}}{n-1}$$

where *n* denotes the number of samplings up to Tmax. There are *n* − 1 intervals that can be utilized to determine *slope_i_* because there are n points between time zero and Tmax.

The weighted average slope (i.e., ASw) is a modified variant of AS that was designed to focus more on early time points, specifically where absorption predominates [20]. The ASw can be calculated by Equation (2):

(2) $${\mathrm{AS}}_{w}=\frac{\sum_{i=1}^{n-1}\left(\frac{Tmax-t_{i}}{Tmax}\;\cdot slope_{i}\right)}{n-1}=\frac{\sum_{i=1}^{n-1}\left(\frac{Tmax-t_{i}}{Tmax}\;\cdot \;\frac{C_{i+1}-C_{i}}{t_{i+1}-t_{i}}\right)}{n-1}$$

The dimensionless quantity (*Tmax* − *t_i_*)/*Tmax* adds more weight to earlier time points where absorption has the most influence, allowing ASw to more closely reflect the absorption process. All of these parameters were estimated in MATLAB^®^ R2022b (MathWorks, Natick, MA, USA) using model-independent approaches.

### 4.4. Simulations

**Figure 8:**
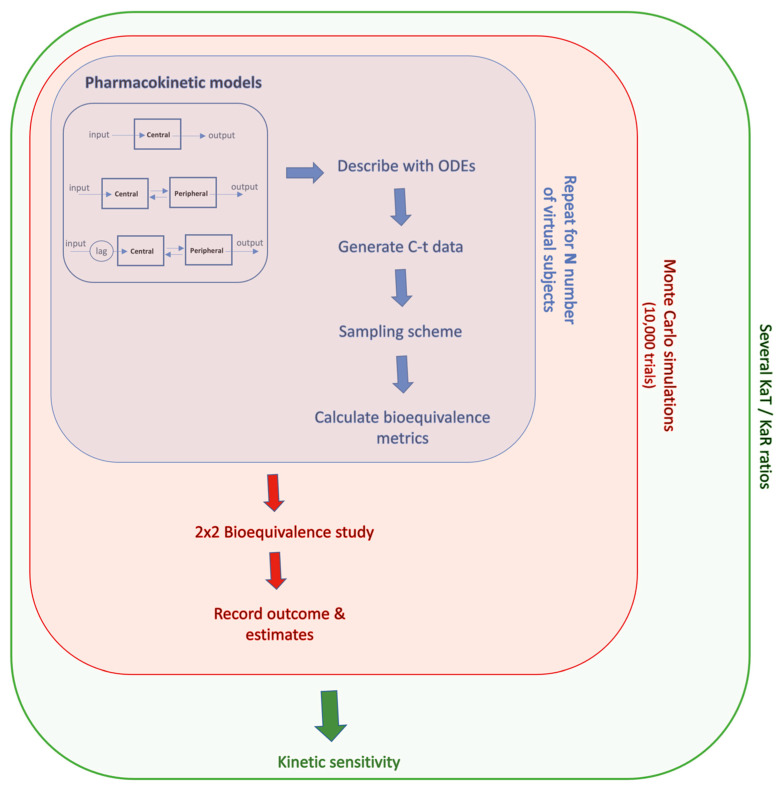
A graphical illustration of the simulation methodology. For each drug, the appropriate pharmacokinetic model was expressed in terms of systems of ordinary differential equations (ODEs), and concentration-time (C-t) data were generated for any number of virtual subjects. The estimated parameters were adapted in a 2 × 2 clinical design, and the whole task was repeated thousands of times using Monte Carlo simulations, for each ratio of the Test/Reference absorption (KaT/KaR).

The modeling methodology developed in this study was initially based on the In Vitro–In Vivo Simulation (IVIVS) approach presented recently [29]. However, significant adaptations were needed, and further modules should have been added in order to: (a) calculate AS and ASw apart from the typical PK parameters; (b) perform iterations for sequential KaT/KaR ratios; and (c) record results for all intermediate estimates at each iteration, in order to allow determination of kinetic sensitivity. An outline of the modeling approach used in this study is shown in Figure 8.

For each drug, the appropriate pharmacokinetic model and parameter estimates were taken from the literature [21,22,23,24,25,26]. The PK parameters of hydrochlorothiazide, donepezil, and amlodipine are listed in Table 2. A one-compartment model with first-order absorption and elimination was employed for amlodipine and donepezil, while a two-compartment model with first-order kinetic transfers and a short lag time in absorption was utilized for hydrochlorothiazide kinetics [21,22,23,24,25,26]. Each model was described in the form of ordinary differential equations by adding appropriately large stochastic errors to each PK parameter. The latter referred to between-subject variability, within-subject variability, as well as residual error.

The simulation methodology continues with the generation of virtual subjects, assuming a statistical distribution (lognormal in this study) [7]. Virtual subjects are generated using the literature estimates for each PK parameter and assuming a statistical distribution (lognormal distribution) (Table 2). Any number of individuals can be simulated, and these virtual subjects are randomly assigned to one of the study’s two groups, receiving either the T or R formulation. For the purposes of this study, the sample size was set equal to 12, 24, and 48 in the case of Monte Carlo simulation studies, while a sample size of 100 was used for the PCA.

A residual error model (proportional, additive, or combined) is used, as well as between- and within-subject variability. The between- and within-subject variabilities for every model parameter were set at 10%. In the case of within-subject variability, two additional levels were explored: a low (5%) and a relatively high (20%). In the simulations for all three drugs, a proportional error model was utilized for the purposes of the current investigation (hydrochlorothiazide, donepezil, and amlodipine) [38]. For each simulated C-t profile, a sampling scheme (Table 3) was used that was tailored to each drug’s pharmacokinetic properties. Based on the sampling technique, the selected C-t data are used to calculate the PK metrics for each of the N virtual patients.

Next, for each participant, all pharmacokinetic measures are appropriately matched to a virtual clinical design [7]; a 2 × 2 crossover design was used in this study. Depending on the clinical design, the regulatory authorities impose the required statistical assessment for BE [1,2]. In the case of the standard 2 × 2 design, the latter includes the logarithmic transformation of AUC and Cmax, the use of ANOVA to determine the residual error and the calculation of the 90% confidence interval around the geometric mean ratio of the BE measure. If the 90% confidence interval falls within the prescribed acceptable criteria (typically 80–125%), bioequivalence is declared [1,2].

The aforementioned approach of simulating a virtual BE study is replicated thousands of times (10,000 trials) using Monte Carlo techniques, and the success or failure of each BE study is determined, along with the geometric mean ratio (GMR) of each parameter, e.g., GMR_AS_ in the case of AS. In addition, the entire procedure is repeated for several KaT and KaR values. In this study, the starting KaT and KaR were both set at 1, implying complete similarity in the average values of T and R. The range of KaT and KaR was from 1.0 to 2.0, with a step of 0.1. This means that differences in the absorption rate up to 100% were explored. After performing all repetitions, the % probability of BE acceptance and GMR estimates of all parameters can be obtained as a function of KaT and KaR.

The entire computational procedure was carried out in MATLAB^®^ R2022b (MathWorks, Natick, MA, USA) by writing the appropriate code. Many methods were used to validate the simulations: (a) by comparing the predicted C-t profiles of the three drugs to the corresponding profiles reported in the literature and those calculated from Simulx (Monolix^®^ 2023R1, Simulation Plus, Lancaster, CA, USA); (b) all scripts were validated sequentially during code development; (c) by comparing the results of each part of the code with other software and the literature; for example, the calculated pharmacokinetic parameters were compared with the estimates from the PKanalix tool (Monolix^®^ 2023R1, Lixoft, Simulation Plus); (d) some parts of the code have already been utilized in the published study for the IVIVS methodology [21]. The numerical solution of the system of ordinary differential equations describing the pharmacokinetic models was implemented through the use of the *ode45* function.

### 4.5. Principal Component Analysis

Machine learning mimics human learning using data and algorithms to improve accuracy [39,40]. “Supervised learning”, “unsupervised learning”, and “reinforcement learning” are the most frequent ML approaches [39,40]. Principal component analysis is a common unsupervised ML method for reducing a high-dimensional set of features [39]. PCA creates a linear combination of the data dimensions to capture as much variability as feasible. Data fluctuates most in the first main component direction, then in the second dimension, and so on.

“Loadings” refer to the contribution of each original variable to the new dimension. The feature impact on this principal component increases as the loading value approaches +1 or −1. The angle between a variable and a principal component shows how a feature contributes to the major component. Thus, loading plots indicate how strongly each attribute impacts a significant component. For the concomitant representation of loadings and scores, the “biplot” is popular. The biplot is a two-dimensional scatter plot with two axes that reflect the two largest explained variance components. In this two-dimensional coordinate system, scores serve as coordinates for the loadings of the first two primary components of each feature. Scree plots are used to select the number of the important principal components. The first component explains the highest proportion of variability, the second a moderate amount, and the third/fourth only a minor amount.

In this study, singular value decomposition was used to reduce the linear dimensionality. It projected data into a lower-dimensional space. Each feature input data were centered and scaled before singular value decomposition. Before conducting the PCA, feature scaling was done because Cmax and AS had different scales. Z-score standardization using “Scikit-learn” *StandardScaler* was used to scale all features [39]. PCA was used with the original number of dimensions to discover connections between all PK metrics. To keep all components, the hyperparameter was set to “none.” A one-dimensional “numpy” array determined each component variation %. The first two primary components were retained after ranking by decreasing variance. The packages “seaborn”, “sklearn”, and “bioinfokit” were used for PCA analysis, and “matplotlib” for statistical visualizations. The entire PCA analysis was implemented in Python v. 3.10.8.

## 5. Conclusions

The idea of “average slope” was recently proposed as a metric to reflect absorption rate and to be used in bioequivalence studies. The aim of this study was to apply a machine learning approach (PCA) and an in silico approach (Monte Carlo PK-BE simulations) in order to explore the properties of Cmax and “average slope” as metrics for absorption rate. Three drugs with different absorption properties were utilized. First, the machine learning approach showed that Cmax does not have the appropriate properties to express absorption rate, since it mainly reflects the extent of absorption. Conversely, average slope (either AS or ASw) exhibits the desired properties to characterize the rate of absorption. Second, the desired kinetic sensitivity of the two new metrics (AS and ASw) was shown and contrasted against the negligible sensitivity of Cmax. Taking also into consideration that Cmax does not have the appropriate units of measurement for being a “rate” measure, this study showed that both in principle and in practice, Cmax fails to characterize the absorption rate. Thus, its wide use only gives a false feeling that the absorption rate is assessed in bioequivalence studies, but this is not what actually happens. Overall, the utilization of modern computational tools showed that Cmax is a completely unsuitable metric for absorption rate, while AS (or ASw) has all appropriate properties both in principle (i.e., kinetic nature, units) and in practice (relationship with absorption rate and kinetic sensitivity). Therefore, if the regulatory authorities wish to assess the “rate”, either AS or ASw can be suitable metrics for expressing the absorption rate.

## Figures and Tables

**Table 1:** The pharmacokinetic properties of the three drugs examined in this investigation.

Characteristic	Drug		
	Hydrochlorothiazide	Donepezil	Amlodipine
**Bioavailability**	60–80%	100%	64–80%
**Tmax**	1–1.5 h	3–4 h	6–12 h
**Model**	Two-compartment	One-compartment	One-compartment
**Lag time**	Yes	No	No
**Elimination half-life**	8–15 h	81.5 +/− 22.0 h	40–50 h
**BCS class**	II	I	I
**Reference(s)**	[21,22,23,24,25]	[26,27]	[28]

**Table 2:** Pharmacokinetic model parameters for the three drugs.

Characteristic	Drug		
	Hydrochlorothiazide	Donepezil	Amlodipine
**Tlag (min)**	24.24	-	-
**Ka (min^−1^)**	0.01288	0.02167	0.01417
**Cl/F (mL/min)**	575	143.33	370
**Q/F (mL/min)**	423.33	-	-
**V1/F (mL)**	137,000	391,000	1300
**V2/F (mL)**	146,000	-	-
**References**	[29]	[19]	[29]

**Table 3:** Sampling schemes used in the in silico methodology for hydrochlorothiazide, donepezil, and amlodipine.

Number	Sampling Times (Minutes)		
	Hydrochlorothiazide	Donepezil	Amlodipine
1	0	0	0
2	10	30	60
3	20	60	120
4	40	90	240
5	60	120	300
6	90	150	360
7	120	180	420
8	180	210	480
9	240	240	600
10	360	360	720
11	480	480	960
12	720	720	1440
13	960	1080	2880
14	1440	1440	4320
15		2160	
16		2880	
17		4320	

## Data Availability

Not applicable.

## Supplementary Materials

The following supporting information can be downloaded at: https://www.mdpi.com/article/10.3390/ph16050725/s1, Calculations.xlsx: An Excel^®^ file for the calculation of AS and ASw.
